# Montelukast Inhibits Platelet Activation Induced by Plasma From COVID-19 Patients

**DOI:** 10.3389/fphar.2022.784214

**Published:** 2022-02-08

**Authors:** Marina Camera, Paola Canzano, Marta Brambilla, G. Enrico Rovati

**Affiliations:** ^1^ Department of Pharmaceutical Sciences, University of Milan, Milan, Italy; ^2^ Centro Cardiologico Monzino IRCCS, Milan, Italy

**Keywords:** COVID-19, leukotrienes, leukotriene antagonists, platelet activation, tissue factor, P-selectin

## Abstract

Leukotrienes are important pro-inflammatory lipid mediators derived from the arachidonic acid metabolism. In particular, cysteinyl leukotrienes, namely LTC_4_, LTD_4_, and LTE_4_ are involved in many of the principal features of asthma, while more recently they have also been implicated in cardiovascular diseases. COVID-19 is characterized by an overwhelming state of inflammation, sometimes resulting in an acute respiratory distress syndrome. Furthermore, severe COVID-19 patients present an endothelial cell damage characterized by a hyperinflammatory/procoagulant state and a widespread thrombotic disease. Leukotriene receptor antagonists, such as montelukast, have long been proven to have an efficacy in asthma, while more recently they have been suggested to have a protective role also in cardiovascular diseases. As elevated levels of LTE_4_ have been detected in bronchoalveolar lavage of COVID-19 patients, and montelukast, in addition to its anti-inflammatory properties, has been suggested to have a protective role in cardiovascular diseases, we decided to investigate whether this drug could also affect the platelet activation characteristic of COVID-19 syndrome. In this contribution, we demonstrate that montelukast inhibits platelet activation induced by plasma from COVID-19 patients by preventing the surface expression of tissue factor (TF) and P-selectin, reducing the formation of circulating monocyte– and granulocyte–platelet aggregates, and, finally, in completely inhibiting the release of TF^pos^-circulating microvesicles. These data suggest the repurposing of montelukast as a possible auxiliary treatment for COVID-19 syndrome.

## Introduction

Leukotrienes (LTs) are important pro-inflammatory lipid mediators derived from the metabolism of the arachidonic acid. In particular, cysteinyl leukotrienes (cysteinyl-LTs), namely LTC_4_, LTD_4_, and LTE_4_ are synthesized *in vivo* by immunocompetent cells such as mast cells, eosinophils, basophils, and monocytes/macrophages (Haeggstrom and Funk, 2011). They are involved in many principal features of asthma, such as bronchoconstriction, hyperresponsiveness, and inflammatory cell recruitment (Sokolowska et al., 2021); more recently, they have also been implicated in other inflammatory conditions, including immune and neurodegenerative disorders, cancer, and particularly in cardiovascular diseases (Capra et al., 2013; Hoxha et al., 2017; Bäck et al., 2019). The biological actions of cysteinyl-LTs are mediated by two officially recognized G-protein-coupled receptors: CysLT_1_ and CysLT_2_. They differ in localization and binding affinities for the different cysteinyl-LTs as well as in their biological activities (Back et al., 2014).

Leukotriene receptor antagonists (LTRAs), such as pranlukast (Onon™), zafirlukast (Accolate™), and montelukast (Singulair™), have long been proven to have an efficacy in asthma therapy. LTRAs have been in use for the last 20 years to treat the airway inflammatory symptoms of mild-to-moderate asthma and allergic rhinitis (Capra et al., 2006; Trinh et al., 2019). In particular, montelukast, the most prescribed drugs among LTRAs in asthma, has been shown to have an excellent safety profile both in adults and children (Virchow and Bachert, 2006).

The SARS-CoV-2 (severe acute respiratory syndrome coronavirus 2) is the cause of the coronavirus disease 2019 (COVID-19) and, while the vast majority of the infected patients range from asymptomatic to mild symptoms, a minority of the patients will eventually develop severe symptoms leading rapidly to hypoxia and acute respiratory distress syndrome (ARDS) requiring hospitalization and oxygen supplementation (Wu and McGoogan, 2020). This severe form is prevalent in the elderly with underlying comorbidities such as hypertension, diabetes, or cardiovascular diseases (Nishiga et al., 2020). COVID-19 is characterized by an overwhelming state of inflammation with an elevated level of circulating chemokines (e.g., MCP-1 and RANTES) and cytokines, such as IL-6, IL-8, and TNF-α eventually leading to a multi-organ dysfunction that has been called COVID-19 cytokine storm syndrome (CSS) (England et al., 2021). Furthermore, an increasing amount of clinical data has documented how SARS-CoV-2 may also predispose patients to endothelial cell damage particularly in the pulmonary vessels (Ackermann et al., 2020), leading to a unique vascular hyperinflammatory/procoagulant state and a widespread thrombotic disease, both in the venous and arterial vascular beds (Teuwen et al., 2020).

In addition to mitigate airway inflammatory symptoms, over the years, a number of *in vitro* and animal studies have suggested LTRAs to have a protective role in cardiovascular diseases (Funk, 2005; Hoxha et al., 2017). Furthermore, in two separate observational studies, a nationwide cohort study in the all Swedish population (Ingelsson et al., 2012) and a retrospective study in 800 asthmatic adults in Albania (Hoxha et al., 2021), exposure to montelukast seems to protect patients from major cardiovascular events. This leads us to speculate on a potential effect of LTRAs on platelet activation, a well-established player in ischemic events. Of note, platelets from COVID-19 patients are characterized by a sustained activation status (Canzano et al., 2021), with cytokines, chemokines, and growth factors released in significantly large amounts upon their stimulation (Taus et al., 2020). Furthermore, we have recently provided evidences that the addition of COVID-19 plasma to plasma-depleted blood from healthy subjects (HS) reproduced the platelet activation, especially in terms of the prothrombotic phenotype, observed *in vivo* in COVID-19 patients (Canzano et al., 2021).

Considering that the elevated levels of LTs have been previously detected in aspirates of patients with ARDS (Sala et al., 1991), while high levels of LTE_4_, a biomarker of total body cysteinyl-LT production, have been detected in bronchoalveolar lavage (BAL) of hospitalized patients with severe COVID-19 syndrome (Archambault et al., 2021), suggesting a role for eicosanoids in the pathological response to SARS-CoV-2 infection, we decided to investigate whether montelukast, in addition to its anti-inflammatory properties, could also affect the expression of the major markers of platelet activation such as tissue factor (TF) and P-selectin as well as the formation of platelet–leukocyte aggregates and microvesicle (MV) release observed in COVID-19 syndrome.

## Methods

### Antibodies and Reagents

Antibodies were obtained from the following sources: mouse anti-human P-selectin APC (CD62P), mouse anti-human CD14 PerCP, mouse anti-human CD41 PerCP Cy5.5, mouse IgG FITC, and mouse IgG APC were from Becton Dickinson; mouse anti-human TF BV421 (clone HTF-1) and mouse anti-human CD41 PE were from Beckman Coulter; and mouse anti-human tissue factor FITC (clone VIC-7) was from Biomedica. Calcein AM was from Invitrogen.

### Patient Selection

The study took advantage from an existing biobank of plasma samples prepared from a cohort of 46 consecutive COVID-19 patients, whose characteristics are reported in Supplementary Table S1 (Canzano et al., 2021).

Patients with a positive SARS-CoV-2 polymerase chain reaction test and requiring oxygen supplementation were included. The criteria for hospital admission were defined as those requiring inpatient care as a result of the severity of illness based on laboratory and radiological parameters as well as clinical findings. Following admission, all patients received supportive care in line with best international practice. Biochemical variables, including inflammatory and thrombotic parameters (IL-6, C-reactive protein (CRP), lactate dehydrogenase (LDH), fibrinogen, D-dimer, and procalcitonin) and arterial blood gas analysis (pO_2_/FiO_2_ ratio and oxygen saturation) were recorded at hospital admission and immediately before or soon after oxygen supplementation, concomitantly with the blood sampling.

### Blood Collection and Plasma Preparation

Whole blood (WB), sampled at initiation of mechanical ventilation or low-flow oxygen therapy, was drawn using a 19-gauge needle without venous stasis into citrate (1/10 volume of 0.129 M sodium citrate)- and K_2_-EDTA-containing tubes (Vacutainer, Becton Dickinson) and processed within 15 min. For citrate plasma preparation, WB was centrifuged at 1,700 g for 10 min at 4°C. Absence of blood cells in plasma samples was evaluated using a cell counter (Sysmex XS-1000i).

### *In Vitro* Studies

To test the effect of COVID-19 patient plasma on platelet activation, three pools made with plasma from 12 patients were prepared (4 patients/pool). The characteristics of the 12 patients are reported in Supplementary Table S1. Blood from healthy subjects (HS, *n* = 4–6, comparable for blood cell counts) was plasma-depleted by centrifuging WB at 1000×g for 10 min, at room temperature (RT). The plasma-free sample obtained after centrifugation was analyzed using a cell counter (Sysmex XS-1000i) to verify that all cellular components were left in the pelleted fraction. Healthy plasma was then replaced with the COVID-19 plasma pools or with autologous plasma as control. To assess the effect of montelukast on cell activation, blood from healthy subjects was preincubated with the drug (at the indicated concentrations) for 30 min, at room temperature. Blood was then centrifuged at 1000×g for 10 min, at room temperature, and plasma was replaced with the COVID-19 plasma pool or with autologous plasma. The effect of LTE_4_ (0.1–30 nM, 15 min, RT) on platelet-associated TF- and P-selectin-expression was also investigated.

### Platelet Activation and Microvesicle Characterization

Circulating cell-associated TF expression, platelet activation markers, and MV release were analyzed by flow cytometry as previously described (Canzano et al., 2021). In brief, WB (5 µL for platelet and 100 µL for leukocyte analysis) was labeled for 15 min at room temperature in the dark with saturating concentration of αTF and αP-selectin MoAbs together with αCD41 and αCD14 or αCD66 to identify platelets, monocytes, and granulocytes, respectively. Leukocyte–platelet aggregates were identified as double positive events for platelet and leukocyte population markers (CD14^pos^/CD41^pos^ or CD66^pos^/CD41^pos^ for monocyte–platelet or granulocyte–platelet aggregates, respectively). The gating strategies are reported in the Supplementary Figure S1. A total of 10,000 CD41 ^pos^ events and 3,000 CD14^pos^ events per sample were acquired on a Gallios flow cytometer (Beckman Coulter). Fluorochrome-conjugated isotype controls were used in order to quantify the background labeling and to set the dot plot quadrant marker for data analysis (Kaluza Analysis software v1.5; Beckman Coulter). The results are reported as percentage ± SD of positive cells.

For  MV  characterization, 50 μL of  WB was diluted in 150 μL of 0.22 µm-filtered buffer [Hepes (10 mM), NaCl (140 mM), and CaCl_2_ (2.5 mM); pH 7.4] containing phe-pro-arg chloromethyl ketone (PPACK, 15 µM)) to prevent clot formation. To identify intact MVs, excluding cell debris, samples were incubated with calcein AM (10 µM) at 37°C in the dark for 25 min followed by the addition of saturating concentrations of αTF and αCD41 MoAbs. Fluorescence minus one (FMO) control was used to correct gating. The samples were immediately analyzed on a Gallios flow cytometer equipped with four solid-state lasers and enhanced wide forward angle light scatter optimized for MV detection. Flow-check Pro Fluorospheres were daily used to monitor cytometer performance. Megamix-FSC Plus beads (0.5, 0.9, and 3 µm) were used to define the analysis gate and BD Trucount tubes^TM^ to have the absolute count of MVs.

### Statistics

The results are expressed as mean ± standard deviation (SD). Continuous variables among groups were compared by repeated measure one-way analysis of variance (ANOVA) followed by Tukey’s multiple comparisons test. A *p*-value of 0.05 was considered statistically significant. Analyses were performed using SPSS statistical package (v9.4).

## Results

**FIGURE 1:**
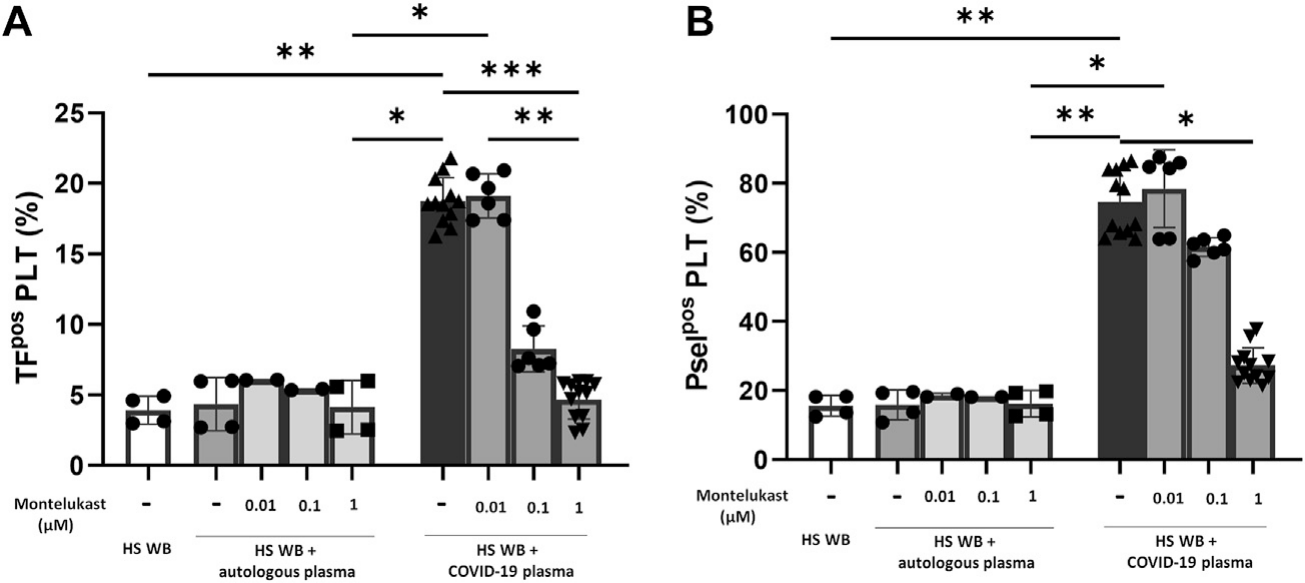
*In vitro* effect of montelukast on platelet-associated tissue factor **(A)** and P-selectin **(B)** expression induced by COVID-19 plasma. Whole blood (WB) from healthy subjects (HS; *n* = 4), pre-incubated with montelukast (0.01–1 µM), was plasma-depleted and reconstituted with COVID-19 plasma pools (*n* = 3 for each HS, dark gray bars) or autologous plasma (light gray bars). The percentage of TF^pos^- and P-selectin^pos^-platelets was assessed by flow cytometry. Data are reported as  mean ± SD. *p < 0.05; **p < 0.01; ***p < 0.001 (TF, tissue factor; Psel, P-selectin; PLT, platelets).

**FIGURE 2:**
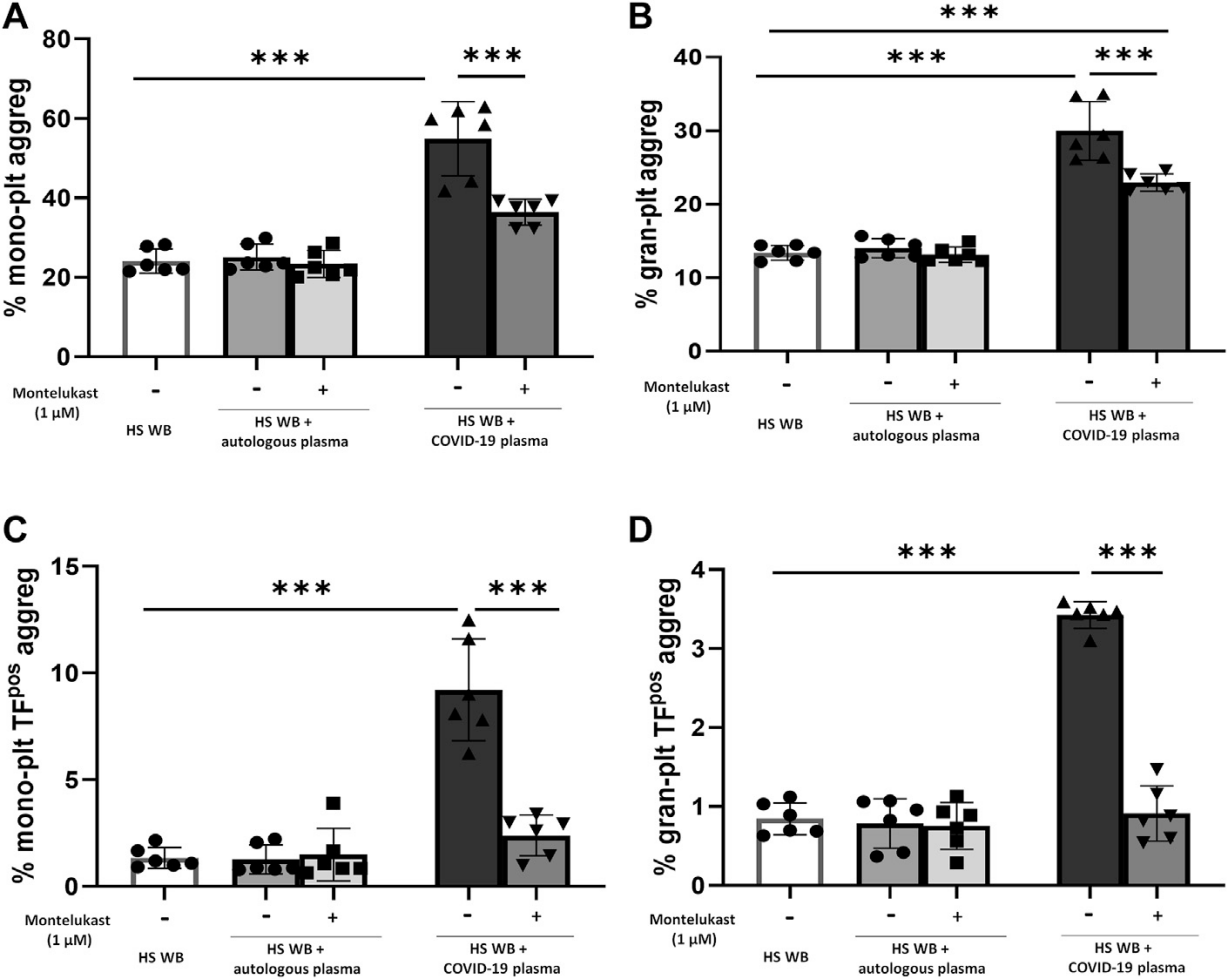
*In vitro* effect of montelukast on platelet–leukocyte aggregate formation induced by COVID-19 plasma. Whole blood (WB) from healthy subjects (HS; *n* = 6), pre-incubated with montelukast (1 µM) as indicated, was plasma-depleted and reconstituted with COVID-19 plasma pools (dark gray bars) or autologous plasma (light gray bars). Total **(A,B)** and TF^pos^ **(C,D)**-platelet–leukocyte aggregates was  measured by flow cytometry. Data are reported as mean ± SD. ***p < 0.001 (TF, tissue factor; plt, platelets; mono, monocytes; gran, granulocytes).

**FIGURE 3:**
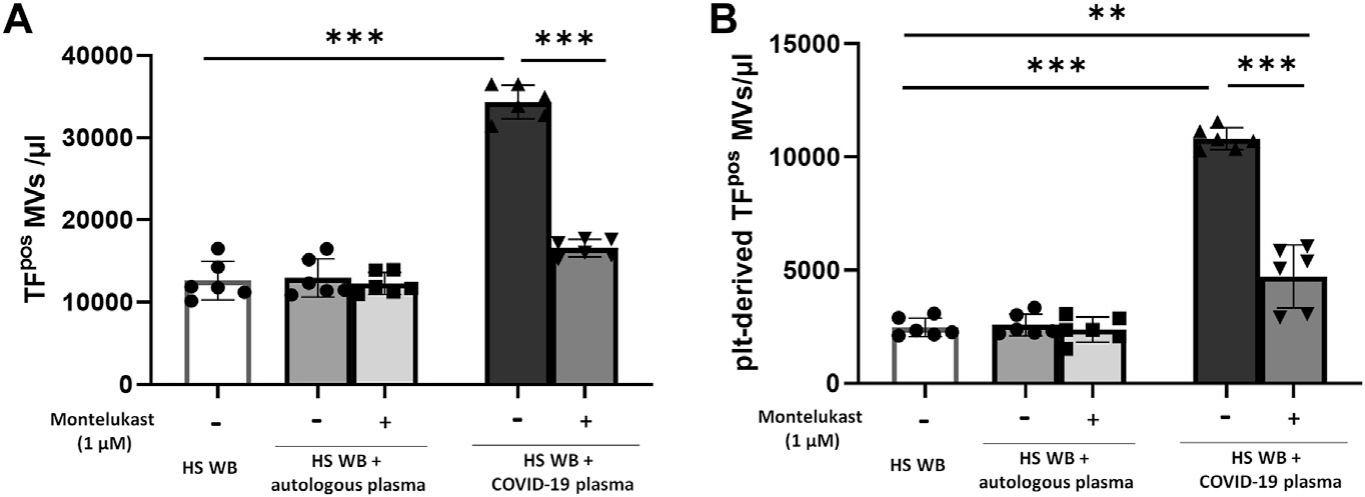
*In vitro* effect of montelukast on microvesicle (MV) release induced by COVID-19 plasma. Whole blood (WB) from healthy subjects (HS; *n* = 3), pre-incubated with montelukast (1 µM) as indicated, was plasma-depleted and reconstituted with the COVID-19 plasma pool (dark gray bars) or autologous plasma (light gray bars). The number of TF^pos^ **(A)** and platelet-derived TF^pos^-MVs **(B)** was measured by flow cytometry. Data are reported as mean ± SD. **p < 0.01; ***p < 0.001 (TF, tissue factor; plt, platelets).

In order to reproduce the platelet activation observed during COVID-19, blood from HS was plasma-depleted and reconstituted with plasma pools from COVID-19 patients or from the same HS blood donors. As expected, plasma from COVID-19 patients significantly increased the number of TF^pos^- and P-selectin^pos^-platelets (Figure 1) and the formation of total and TF^pos^ platelet–monocyte and platelet–granulocyte aggregates (Figure 2) as well as the release of total and platelet-derived TF^pos^ circulating MVs (Figure 3), highlighting the marked prothrombotic phenotype associated with both TF-bearing cells and MVs.

Interestingly, preincubation of HS platelets with montelukast concentration dependently prevented the induction of TF^pos^- and P-selectin^pos^-platelets by COVID-19 plasma, being both completely inhibited at 1 µM (Figure 1). It also significantly reduced the effect of COVID-19 plasma on the formation of circulating monocyte–platelet and granulocyte–platelet aggregates (Figure 2, panel A and B), decreasing the number of those TF^pos^ by 4-times (Figure 2, panel C and D). Finally, montelukast was effective in completely preventing the release of TF^pos^-circulating MVs induced by COVID-19 plasma, reducing by more than 2-times those derived from platelets (Figure 3).

**FIGURE 4:**
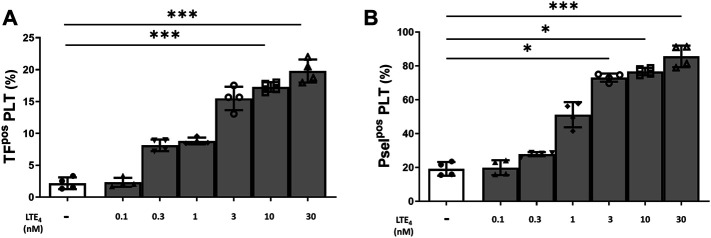
*In vitro* effect of LTE_4_ on platelet-associated tissue factor **(A)** and P-selectin **(B)** expression. Whole blood was stimulated with LTE_4_ (0.1–30 nM), for 15 min at room temperature. The percentage of TF^pos^- and P-selectin^pos^-platelets is shown. Data are reported as mean ± SD. *p < 0.05; ***p < 0.001 (TF, tissue factor; Psel, P-selectin; PLT, platelets).

Overall, these data point to an involvement of LTs in the platelet activation induced by COVID-19 plasma. Indeed, *in vitro* stimulation of platelets from HS with LTE_4_, the most stable of cysteinyl-LTs (Sokolowska et al., 2021), concentration dependently induced TF and P-selectin expression (Figure 4).

## Discussion

The present study reports, for the first time, that montelukast significantly inhibits platelet activation induced by plasma from COVID-19 patients, suggesting its use as a possible auxiliary treatment in this syndrome.

There is now a wide consensus in the literature that COVID-19 is associated not only with a CSS characterized by an hyperimmune or hyperinflammatory response (England et al., 2021) but also with endothelial cell damage ultimately leading to micro- and macro-thrombosis in the pulmonary vessels as well as in other organs (Ackermann et al., 2020; Teuwen et al., 2020). Despite several different drugs/strategies are available to treat COVID-19 patients today, from antiviral drugs to SARS-CoV-2-neutralizing antibodies, from IL-6 receptor monoclonal antibodies to corticosteroids or aspirin, and while several new approaches are tested in ongoing clinical trials, an unanimous consensus to treat this disease has not yet been achieved (COVID-19 Treatment Guidelines Panel, NIH. Available at https://www.covid19treatmentguidelines.nih.gov/).

Cysteinyl-LTs, in addition to being the most potent broncho-constrictors known in man, have a well-recognized role in immune cell and macrophages activation as well as cytokine (IL-6, IL-8, TNF-α, and MIP-1β) or chemokine (MCP-1 and RANTES) release (Back et al., 2014). Accordingly, LTRAs significantly inhibited pro-inflammatory cytokine production mostly through inhibition of NF-kB (Maeba et al., 2005; Tahan et al., 2008), a transcription factor that is known to control several genes involved in inflammation including IL-6, IL-8, and TNF-α, all of which would enhance the hyperimmune/inflammatory response. Of note, the severity of pulmonary complications in COVID-19 seems to be closely related to IL-6 and TNF-α peak levels (Chen et al., 2020). For these reasons, largely speculative until now, eicosanoids have been hypothesized to be involved in various aspects of COVID-19 pathology (Arnardottir et al., 2020; Hammock et al., 2020). In particular, the use of montelukast has been proposed as a possible therapy, particularly due to its anti-inflammatory activities (Aigner et al., 2020; Barré et al., 2020; Funk and Ardakani, 2020; Sanghai and Tranmer, 2020). Accordingly, the first phase III clinical trial testing the cysteinyl-LT receptor antagonist montelukast in COVID-19 patients has already been planned (https://clinicaltrials.gov/ct2/show/NCT04389411).

Despite LT levels cannot be reliably measured in plasma (Heavey et al., 1987; Sokolowska et al., 2021), as mentioned before, the elevated levels of LTE_4_, the only sufficiently stable LT to be prominent in biologic fluids (Sokolowska et al., 2021), have been detected in BAL of patients with severe COVID-19 (Archambault et al., 2021), while elevated levels of LTs have been previously detected in aspirates of patients with ARDS (Sala et al., 1991). Here we demonstrate that, indeed, exogenous LTE_4_ is able to induce expression of known markers of platelet activation at concentrations comparable to those obtained in BAL of COVID-19 patients (Archambault et al., 2021). Thus, the data presented here suggest that montelukast, if administered in the early phase of the disease, not only has the potential to limit the acute and chronic lung tissue damage in COVID-19 patients by treating the hyperimmune/hyperinflammatory response and taming the CSS, but it may also limit the massive platelet activation and prothrombotic phenotype, thus serving as a potential “single” approach for the two most prominent aspects of COVID-19 syndrome. To this point, two very recent retrospective analyses demonstrated that either asthmatic patients receiving montelukast had fewer episodes of confirmed COVID-19 or experienced significantly fewer events of clinical deterioration (Bozek and Winterstein, 2020; Khan et al., 2021).

Another interesting aspect may involve the thrombotic complications of SARS-CoV2 vaccination. In fact, while the exact mechanism by which adenovirus-vectored COVID-19 vaccines trigger the vaccine-induced immune thrombotic thrombocytopenia (VITT) is still unclear; this syndrome is thought to involve a FcγRIIA receptors-dependent platelet activation causing platelet P-selectin’s expression, secretion of alpha granules, and release of procoagulant MVs, leading to TF accumulation into developing thrombi (Marchandot et al., 2021). Therefore, montelukast treatment might also help to prevent or mitigate this rare, but serious adverse event upon vaccination.

At this stage, we can only speculate on the mechanism of antiplatelet activity of montelukast. While it is known that human platelets express both CysLT_1_ and CysLT_2_ receptors, and that pranlukast inhibits cysteinyl-LT-induced RANTES release (Hasegawa et al., 2010), contrasting data are present in the literature on platelet activation by LTs (Austen et al., 2009; Foster et al., 2013). While the results of our *in vitro* experiments showing that LTE_4_ concentration dependently induced TF and P-selectin expression suggest that LTE_4_ itself plays a central role in the described platelet activation, we cannot rule out the involvement of other players. Of note, in our model, IL-6, the expression of which can be modulated by montelukast, at concentrations comparable to those found in COVID-19 patients, significantly potentiated the effect of low-concentration adenosine diphosphate (ADP) or thromboxane A_2_, and accordingly, aspirin and the P2Y_12_ inhibitor AR-C69931MX prevented platelet activation induced by COVID-19 plasma (Canzano et al., 2021). Therefore, considering that montelukast has been shown to have some off-target effects, namely inhibition of some P2Y receptors (Mamedova et al., 2005; Woszczek et al., 2010), the data presented here might be either due to inhibition of its primary target or to a secondary target directly involved in platelet activation.

In conclusion, we believe that our data, highlighting the possible contribution of montelukast not only to mitigate the CSS but also in alleviating the peculiar platelet prothrombotic phenotype of COVID-19, should foster the scientific community to further consider the repurposing of montelukast, or other approved LTRAs, as an innovative strategy for COVID-19 syndrome.

## Data Availability

The raw data supporting the conclusion of this article will be made available by the authors, without undue reservation.

## Abbreviations

LTs, leukotrienes; cysteinyl-LTs, cysteinyl leukotrienes; LTRAs, leukotriene receptor antagonists; SARS-CoV-2, severe acute respiratory syndrome coronavirus 2; COVID-19, coronavirus disease 2019; ARDS, acute respiratory distress syndrome; CSS, cytokine storm syndrome; BAL, bronchoalveolar lavage; TF, tissue factor; MV, microvesicle.
