# Supplementary material for: Montelukast Inhibits Platelet Activation Induced by Plasma From COVID-19 Patients
Source: Front Pharmacol. 2022 Feb 8;13:784214. doi: 10.3389/fphar.2022.784214 (PMC8863130; doi:10.3389/fphar.2022.784214)
Supplement: Supplementary file 1 [file Table1.DOCX]

Table S1. Demographic and Clinical Characteristics of Enrolled COVID-19 Patients

| Characteristic | All  (n=46) | Included  (n=12) | p value |
| --- | --- | --- | --- |
| Age —yr | 72 [58-84] | 70 [62-76] | 0.727 |
| Male sex—no. (%) | 28 (61) | 10 (83) | 0.186 |
| In hospital mortality | 10 (21.7) | 4 (33.3) | 0.457 |
| Length of hospitalization | 40.5 [29-43.5] | 35 [30-41] | 0.478 |
| Interleukin–6 (pg/mL) | 41 [27-77] | 17 [11-38] | 0.449 |
| CRP (mg/dL) | 8.8 [4.6-12.3] | 2.3 [0.9-5.3] | 0.989 |
| D-Dimer (µg/L) | 1422 [774-2139] | 740 [653-1467] | 0.996 |
| *Hemogas analysis* |  |  |  |
| Oxigen saturation | 94.5 [92-97] | 92 [88-95] | 0.331 |
| Respiratory rate | 20 [18-24] | 22 [20-25] | 0.977 |
| pCO2 | 36.5 [33-44] | 36 [32-38] | 0.625 |
| pO2/FiO2 ratio | 177 [132-343] | 166 [124-254] | 0.558 |
| pH | 7.5 [7.4-7.5] | 7.5 [7.46-7.52] | 0.755 |
| Lactate | 1.3 [0.9-1.6] | 1.4 [1.2-1.5] | 0.709 |
| *Cardiovascular risk factors* |  |  |  |
| Smoking status |  |  |  |
| Active smoker—no. (%) | 3 (8) | 1 (8) | 1 |
| Former smoker—no. (%) | 6 (16) | 4 (33) | 0.190 |
| Dyslipidemia—no. (%) | 2 (5) | 1 (8) | 0.508 |
| Hypertension—no. (%) | 32 (69) | 8 (67) | 1 |
| Diabetes—no. (%) | 4 (11) | 1 (8) | 1 |
| *Past medical history* |  |  |  |
| Cardiovascular diseases—no. (%) | 33 (87) | 11 (92) | 0.258 |
| Cerebrovascular diseases—no. (%) | 7 (18) | 3 (25) | 0.412 |
| Respiratory diseases—no. (%) | 10 (27) | 1 (8) | 0.428 |
| Endocrinological diseases—no. (%) | 8 (21) | 3 (25) | 0.681 |
| Renal diseases—no. (%) | 2 (5) | 0 | 1 |
| *Pharmacological therapy* |  | |  |
| LMWH heparin treatment | 36 (80) | 7 (58) | 0.264 |
| LMWH heparin dose |  |  |  |
| 0 | 9 (20) | 5 (42) | 0.138 |
| 4000U | 1 (2.2) | 0 |  |
| 6000U | 5 (11.1) | 1 (8) |  |
| 4000U x2 | 17 (37.8) | 2 (17) |  |
| 6000U x2 | 11 (24.4) | 4 (33) |  |
| 8000U x2 | 2 (4.4) | 0 |  |
| Antiplatelets—no. (%) | 5 (11) | 2 (17) | 0.626 |
| Hydroxychloroquine—no. (%) | 4 (12) | 1 (8) | 1 |
| Cortison—no. (%) | 13 (30) | 1 (8) | 0.258 |
| *Biochemical parameters* |  |  |  |
| Ferritin (µg/L) | 857 (498-1686) | 863 [433-1242] | 0.830 |
| AST (U/L) | 35 [23-44] | 20 [16-27] | 0.464 |
| ALT (U/L) | 30 [20-57] | 32 [24-45] | 0.497 |
| LDH (U/L) | 326 [249-383] | 193 [162-252] | 0.437 |
| S-Creatinine (mg/dL) | 0.9 [0.7-1] | 0.83 [0.6-1] | 0.843 |
| PT/ INR | 1.22 (1.15-1.3) | 1.2 (1.15-1.24) | 0.471 |
| PTT ratio | 1.35 [1.18-1.47] | 1.3 [1.2-1.5] | 0.577 |
| TN T (ng/L) | 26 [13-48] | 14.5 [11-34.3] | 0.388 |
| pBNP (ng/L) | 197 [97-315] | 165 [69-437] | 0.995 |
| Procalcitonin (ng/mL) | 0.1 [0.1-0.2] | 0.09 [0.05-0.17] | 0.727 |
| Fibrinogen (mg/dL) | 546 [412-637] | 408 [395-436] | 0.998 |
| *Blood cell count* |  |  |  |
| WBC (10^3/uL) | 10.1 [6.6-12.3] | 7.8 [6-12.7] | 0.678 |
| NEUT# (10^3/uL) | 6.8 [4-9.8] | 5.4 [4.4-9.6] | 0.637 |
| LYMPH# (10^3/uL) | 1.3 [0.9-2.1] | 1.2 [0.8-2.1] | 0.503 |
| MONO# (10^3/uL) | 0.7 [0.5-0.9] | 0.8 [0.6-0.9] | 0.407 |
| RBC (10^6/uL) | 3.7 [3.2-4] | 3.5 [3.2-4.1] | 0.836 |
| HGB (g/dL) | 10.7 [9.8-11.9] | 10.9 [9.5-12.7] | 0.719 |
| HCT (%) | 33.8 [30.9-36.9] | 33.4 [28.8-39] | 0.979 |
| PLT (10^3/uL) | 267 [202-314] | 256 [199-340] | 0.909 |
| PDW (fL) | 12.9 [11.6-14.2] | 13.9 [11.8-15.7] | 0.287 |
| MPV (fL) | 11 [10.4-11.6] | 11.4 [10.8-12] | 0.174 |
| P-LCR (%) | 32.5 [27.2-36.3] | 35.9 [30.2-40.9] | 0.159 |
| IPF (%) | 3.7 [2.7-5.7] | 4.4 [3-7.4] | 0.188 |
