# Supplementary material for: Montelukast Inhibits Platelet Activation Induced by Plasma From COVID-19 Patients
Source: Front Pharmacol. 2022 Feb 8;13:784214. doi: 10.3389/fphar.2022.784214 (PMC8863130; doi:10.3389/fphar.2022.784214)
Supplement: Supplementary file 2 [file Image1.PDF]

Supplementary Figure SI

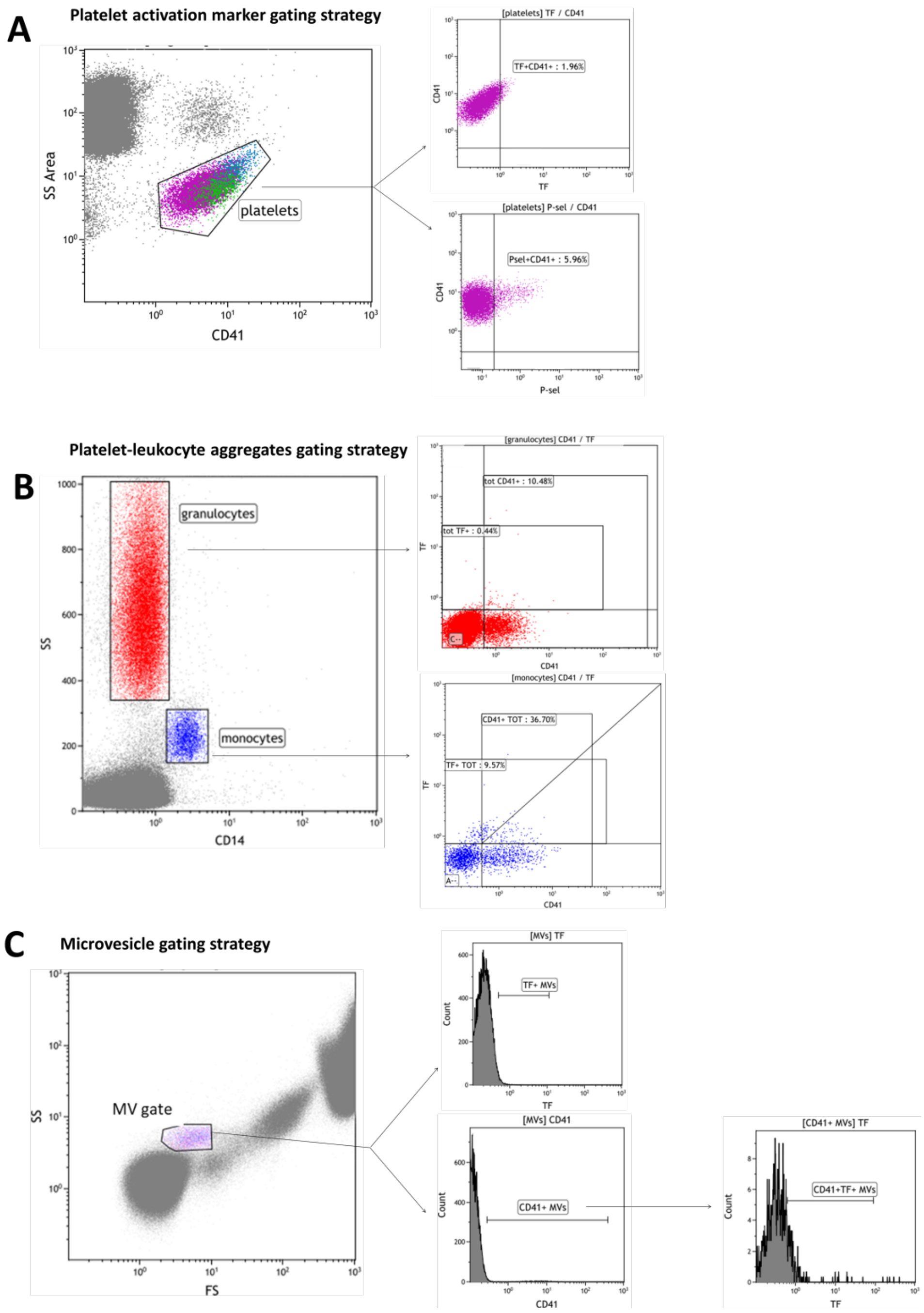

Supplementary Figure SI. Gating strategy for flow cytometry analysis of platelet activation markers (A), platelet-leukocyte aggregates (B) and microvesicles (C). (SS = side scatter; FS = forward scatter; TF = tissue factor; Psel = P-selectin; MV = microvesicles).
